# DapBCH: a disease association prediction model Based on Cross-species and Heterogeneous graph embedding

**DOI:** 10.3389/fgene.2023.1222346

**Published:** 2023-09-22

**Authors:** Wanqi Shi, Hailin Feng, Jian Li, Tongcun Liu, Zhe Liu

**Affiliations:** ^1^ School of Mathematics and Computer Science, Zhejiang A & F University, Hangzhou, Zhejiang, China; ^2^ College of Media Engineering, Zhejiang University of Media and Communications, Hangzhou, Zhejiang, China

**Keywords:** heterogeneous network, biological information, comorbidity, cross-species, multiple networks

## Abstract

The study of comorbidity can provide new insights into the pathogenesis of the disease and has important economic significance in the clinical evaluation of treatment difficulty, medical expenses, length of stay, and prognosis of the disease. In this paper, we propose a disease association prediction model DapBCH, which constructs a cross-species biological network and applies heterogeneous graph embedding to predict disease association. First, we combine the human disease–gene network, mouse gene–phenotype network, human–mouse homologous gene network, and human protein–protein interaction network to reconstruct a heterogeneous biological network. Second, we apply heterogeneous graph embedding based on meta-path aggregation to generate the feature vector of disease nodes. Finally, we employ link prediction to obtain the similarity of disease pairs. The experimental results indicate that our model is highly competitive in predicting the disease association and is promising for finding potential disease associations.

## 1 Introduction

It is important to understand the association between diseases for diagnosing, curing, and taking precautions against diseases (Jin et al., 2019). Since there may be direct or indirect causal relationships between diseases, patients experience multiple diseases at the same time. For example, end-stage renal disease (ESRD) is frequently present in HIV-1 patients, while chronic obstructive pulmonary disease (COPD) is often accompanied by lung cancer, osteoporosis, cachexia, and cardiovascular disease (Mlynarski et al., 2005; Decramer and Janssens, 2013). In addition, the tumor-related disease always indicates serious complications, which has a high comorbidity pattern with hypertension, respiratory diseases, and cerebrovascular diseases (Ko et al., 2016). Moreover, the main cause of death in patients with cirrhosis is hepatocellular carcinoma (HCC) (Ji et al., 2015). Currently, numerous treatment strategies have been investigated in relation to various diseases (Shao et al., 2013). However, the current medical research on comorbidity is not perfect, and there is still great uncertainty in the association between many diseases. Therefore, how to effectively analyze the relationship between diseases and discover potential comorbidity relationships has become a new research issue.

Previously, people employed traditional biological experimental methods to explore the association between diseases. It requires a lot of human resources and financial support, for the reason that these methods need to target a great quantity of genes to identify related diseases (Uffelmann et al., 2021). Fortunately, with the large-scale integration of various experimental data such as human gene annotations, disease phenotypes, and protein–protein interaction, data support has been provided to better elucidate the underlying biological mechanisms of complex diseases (Silverman et al., 2020; Graw et al., 2021). Although these biological data are complex, it becomes simple and clear to represent various biological data forms through the network, so network-based approaches have emerged as the main prediction method for the association between diseases (Wang et al., 2011; Ji et al., 2019; Yue et al., 2020).

The network-based models are based on the hypothesis of ‘guilt-by-association,’ where genes close to each other in physiology or functionally often participate in the same biological pathway (Aravind, 2000; Oliver, 2000). Since measuring the distance between candidate genes and known disease genes in the protein–protein interaction (PPI) network is a crucial component of the network-based model, numerous computerized methods have been devised (Sharan et al., 2007; Shlomi et al., 2008). Goh et al. (2007) built a network that links two different genes together if they are linked to the same disease. Tian et al. (2008) reconstructed a network by adding protein interactions, gene interactions, and gene expression correlations to calculate more accurate distances between genes. Ulitsky and Shamir (2007) further supplemented this by adding interactions from human cell cycle networks and yeast two-hybrid experiments. The aforementioned three models all utilize the direct protein interaction, but they all suffer from insufficient network data. Based on the assumption that diseases with an overlapping phenotype have potentially functionally similar genes, Wu et al. (2008), Li and Patra (2010), and Vanunu et al. (2010) have found that combining disease phenotype networks and PPI networks in priority tasks would result in better performance of the model. Wu et al. (2008) employed the linear regression method CIPHER to predict the disease gene associations by combining the PPI network and phenotype network, and analyze the relevance between the distribution of phenotypic similarity and gene compact distribution in the protein interaction network. Li and Patra (2010) proposed the RWRH model to apply the RWR algorithm to the heterogeneous network they constructed, connecting gene networks and phenotype similar networks using known gene phenotype associations. Luo and Liang (2015) further developed RWRHN on the basis of the RWRH algorithm, enabling it to carry out random walks on heterogeneous networks to predict potential candidate genes for genetic diseases. RWRHN is a random walk algorithm with resistance, and its main contribution is to predict the background of protein network reconstruction through linking, so as to obtain a more reliable PPI network. The heterogeneous network can describe the real world more precisely compared to homogeneous graphs, so more meta-pathway-based models (Jin et al., 2019; Xiong et al., 2019; Yang et al., 2019; Zhang et al., 2019; Deng et al., 2020; Zhang et al., 2020; Zhou et al., 2020; Ata et al., 2021; He et al., 2021) have been developed to better adapt to heterogeneous biological networks. Luo et al. (2016) proposed the RMLM and RMMSe methods for mining meta-path-based miRNA–target interactions by constructing networks. Jin et al. (2019) constructed a heterogeneous network by integrating the disease–gene association network, miRNA–gene association network, gene–disease association network, and protein–protein interactions networks, and then infer disease association by applying random walk and skip-gram based on the meta-path. Compared with the aforementioned methods, the Metapath2Vec algorithm they adopted can better preserve the structural and semantic interrelationships. However, the application of this method is limited by the existing large differences between vertexes or link attributes in heterogeneous networks. With the success of network embedding methods in analyzing various networks, researchers are increasingly using heterogeneous network representation learning methods to extract node (edge) embeddings in biological networks in order to more fully extract information from heterogeneous biological networks and thus obtain better disease association prediction performance. Yang et al. (2018) proposed an embedded representation model HerGePred based on the heterogeneous disease gene-related network. This model restarts random walk on the reconstructed heterogeneous disease gene network and obtains the low-dimensional vector representation of nodes in the network, which improves the prediction performance. Altabaa et al. (2022) proposed and evaluated the geneDRAGNN method using graph neural networks, which uses information from gene–gene interaction networks to predict disease associations.

Although the aforementioned model in the prediction of disease association study has made great progress, but there are still some limitations. Some of the models previously mentioned (Goh et al., 2007; Ulitsky and Shamir, 2007; Tian et al., 2008; Wu et al., 2008; Li and Patra, 2010; Vanunu et al., 2010) and their similar ones (Suratanee and Plaimas, 2015; Zhang et al., 2016; Iida et al., 2020) only employ protein–protein interactions and related genes to predict, so they will suffer from insufficient data. There are also some models (Jin et al., 2019; Zhang et al., 2019; Zhou et al., 2020; Ata et al., 2021) that ignore the content of nodes, or other models (Deng et al., 2020; He et al., 2021), only to consider both ends of the meta-paths and ignore the intermediate nodes of each meta-path, resulting in the loss of information on heterogeneous graphs. Moreover, some models (Xiong et al., 2019; Zhang et al., 2020) rely on a single meta-path to gain the target node’s embedding in the heterogeneous graph, which may lose information about other meta-paths and lead to sub-optimal performance.

In this paper, we develop a disease association prediction model, which is based on a cross-species heterogeneous biological network and a heterogeneous graph embedding method by applying the meta-path-aggregated graph neural network (DapBCH). Our model has the following contributions.

1. We construct a cross-species heterogeneous network to alleviate the problem of insufficient data. The phenotype of model organisms can be applied to human phenotype studies (Liao and Zhang, 2008). Therefore, we apply mice as model organisms and effectively integrate their biological data into heterogeneous bioinformatics networks. Specifically, DapBCH combines the human disease–gene network, mouse gene–phenotype network, human–mouse homologous gene network, and human protein–protein interaction network to create a more complete bioinformatics network.
2. We apply a heterogeneous graph embedding method (MAGNN) (Fu et al., 2020) to extract the features of disease nodes, which can fully capture the node content and context structure of the heterogeneous biological network. Specifically, first, we project nodes of different dimensions into the same vector space to address the issue of various node types in heterogeneous biological networks. Second, we apply the attention mechanism aggregation in each meta-path to handle the problem that the aforementioned methods (Jin et al., 2019; Zhang et al., 2019; Deng et al., 2020; Zhou et al., 2020; Ata et al., 2021; He et al., 2021) only consider the neighbor nodes in the meta-path, while information about phenotype-related genes that are not connected to the target disease node is ignored. Third, we aggregate the potential vectors obtained from the four meta-paths to obtain the final node embedding, which tackles the problem of relying only on a single meta-path in the heterogeneous biological network and not making full use of various biological paths.
3. Furthermore, we scientifically verify the predicted comorbidities by reviewing the literature, demonstrating that DapBCH is effective in predicting disease associations.

Experiments have demonstrated that DapBCH can more accurately predict disease associations. Ablation experiments confirm the correctness of our method, that is, adding mouse phenotype association data and human–mouse homologous gene data, as well as selecting multiple meta-pathways, can improve the accuracy of disease association prediction. Moreover, scientific validation of our prediction of comorbidity demonstrates that our model can detect potential disease associations.

## 2 Materials and methods

### 2.1 Biological data

The main data used in this paper include the following: (1) the association between human diseases and genes; (2) mouse–gene phenotype association; (3) human–mouse homologous gene; (4) protein interaction group; and (5) a set of known positive disease–disease associations. Among them, the first three groups of data are obtained from the MGI database (Eppig, 2017), and the fourth group of data is obtained from the STRING database (Mering et al., 2003). The fifth dataset is obtained by integrating three manually checked datasets (Pakhomov et al., 2010; Suthram et al., 2010; Mathur and Dinakarpandian, 2012; Žitnik et al., 2013; Cheng et al., 2014) of disease pairs with high similarity. The specific dataset of this experiment also includes the mapping set of EntrezGene ID to GO ID for human gene, the score link dataset of human gene EntrezGene ID to mouse gene MGI ID, and the relationship between human gene GO to protein ID. The sources of all datasets are as follows:

Human disease and gene association: in this experiment, the association dataset from the mouse genome information (MGI) database is derived from MGI_DO.rpt.txt.

Mouse–gene phenotype association: in this experiment, as the mapping set from the mouse gene MGI ID to phenotype, it is derived from MGI_GenePheno.rpt of the MGI database.

Human–mouse homologous gene: in this experiment, the human gene EntrezGene ID is mapped to the mice gene MGI ID, and the data are obtained from HMD_HumanPhenotype.rpt of the MGI database.

A set of known positive disease–disease associations: in this experiment, one of the datasets that we integrated is from the linked disease pairs obtained by fusing molecular data by Žitnik et al. (2013). Another dataset is the confirmed similar disease pairs collected by Cheng et al. (2014). The last part of the dataset is the disease pairs extracted by Mathur and Dinakarpandian (2012) through literature validation. There are 73 diseases and 92 disease–disease associations in this collection of known disease associations.

Idmapping_selected.Tab: in this experiment, as the mapping set from EntrezGene ID to GO ID for human genes, it comes from the database of Georgetown University in the United States (Huang et al., 2003).

Protein interaction group: in this experiment, as the association dataset of human genes and proteins, it comes from the Final_GO_ProteinID_human.txt of the Gene Ontology Resource database (Consortium, 2004).

9606.protein.links.v11.5.txt: in this experiment, as the score link dataset of the protein interaction, it is derived from the STRING database.

### 2.2 Methods

Our model, DapBCH, constructs a heterogeneous biological network cross-species and applies the heterogeneous graph embedding method (MAGNN) to predict disease association. The key steps of this model are as follows: (1) network construction: apply the aforementioned biological data to generate node information and adjacency matrix, and then construct a heterogeneous graph neural network. The heterogeneous network includes human–disease gene, mouse–phenotype gene, human–mouse homologous gene, and protein–protein interactions; (2) heterogeneous graph embedding: first, we convert the contents of different types of nodes into the vector space of the same dimension, then we apply intra-meta-path aggregation, and finally apply inter-meta-path aggregation on the four meta-paths to generate the feature vector of disease nodes; and (3) network-based disease association prediction: apply link prediction using the acquired disease node feature vectors to obtain the similarity of disease pairs.

#### 2.2.1 Network construction

We construct a heterogeneous biological network by combining four different networks: (a) human disease–gene association network, (b) mouse gene–phenotype association network, (c) human–mouse homologous gene network, and (d) the human protein–protein interactions network. We list the contents of each network of the heterogeneous biological network in Table 1.

**TABLE 1:** Description of each network of the heterogeneous biological information network.

Network	Node		Association	Source
Human disease–gene network	Human diseases	2,958		
	Human genes	3,562	3,687	MGI
Mouse gene–phenotype network	Mouse genes	12,319		
	Mouse phenotype	8,801	77,644	MGI
Human–mouse homologous gene network			10,491	MGI
Human protein–protein interaction network	Proteins (genes)	13,281	229,524	STRING

Human disease–gene association network. We collect the experimentally validated human disease–gene associations from the MGI database. The genes are annotated using the Entrez IDs, and the diseases are represented using their OMIM identifier. The human disease–gene associations in database are picked out for providing information on humans. We artificially map Entrez to DO terms and annotate each DO term with its associations. Finally, we obtain a total of 3,687 gene–disease associations, linking 2,958 human diseases to 3,562 human genes.

Mouse gene–phenotype association network. We collect mouse gene–phenotype associations from the MGI database. Because our evaluation set uses human gene identifiers, we use human–mouse homologous genes to connect our mouse phenotypes to the network.

Human–mouse homologous gene network. We collect the human and mouse gene associations from HMD_HumanPhenotype.rpt in the MGI database, which include the mouse orthologs of human genes and human orthologs of mouse genes. We map each mouse gene to their human direct homologs and obtain 10,491 human genes, where the mouse direct homologs have phenotype associations.

The human protein–protein interaction network. Physical protein–protein interactions are extracted from the STRING database. We artificially map protein to GO terms based on Idmapping_selected.Tab, Final_GO_ProteinID_human.txt, and 9606.protein.links.v11.5.txt. We map the proteins to Idmapping_selected.Tab and screen out these entries that are not mapped to the database to obtain the desired association table of protein interactions relevant to this experiment. Furthermore, we extract the confidence score of the interaction group from the STRING database and delete the interactions with confidence less than 400. The protein–protein interaction network we obtained consists of 13,281 proteins and 229,534 interactions. For the extracted directly connected protein pairs, their corresponding coding genes are connected by unweighted edges in the PPI network, and we set the weight to 1.0.

**FIGURE 1:**
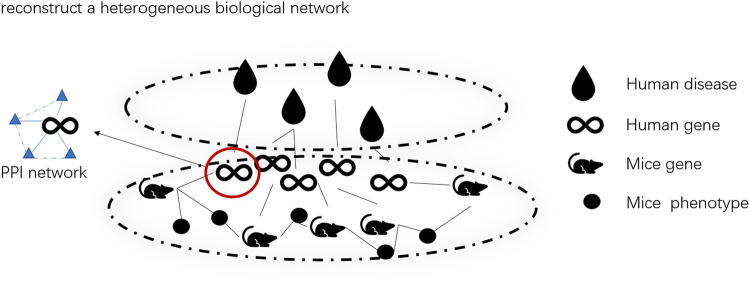
Heterogeneous biological network by assembling four types of networks.

The heterogeneous biological network across species we built is shown in Figure 1.

#### 2.2.2 MAGNN

MAGNN is a graph neural network based on the meta-path for heterogeneous graph embedding. It mainly includes three steps: node content transformation, intra-meta-path aggregation, and inter-meta-path aggregation to generate node embedding. A key assumption of our model is that diseases (genes or phenotypes) that are physically or functionally close to each other in the network have higher similarity. If these two diseases are related, the genes related to these two diseases should be close to each other in the gene phenotype network or the protein network. This allows us to depend on the existing edges to dig unknown disease-related associations. Therefore, we select four meta-paths, namely, M1 (disease → human gene → disease), M2 (disease → human gene → human gene → disease), M3 (disease → human gene → mouse gene → human gene → disease), and M4 (disease → human gene → mouse gene → phenotype → mouse gene → human gene → disease), for extraction of disease node embeddings.

#### 2.2.3 Heterogeneous graph embedding

Different network embedding techniques have been proposed for extracting embeddings in complex network structures (Li et al., 2015; Hamilton et al., 2017; Vaswani et al., 2017; Shi et al., 2018). Most of the existing network embedding methods can only be applied to homogeneous networks, where both node types and edge types are the same in their networks. However, in order to describe the disease association network more realistically and accurately, different genes and phenotypes are integrated into biological networks with different features in our work. Therefore, we need to first project node features of different types (e.g., disease and phenotype) in heterogeneous biological networks into the same latent vector space. Specifically, we designed a parametric weight matrix *W*. For a node $d\;\in \;D_{A}$ of type *A* ∈ **A**, we obtain

$$h_{d}^{\prime }=W_{A}\cdot x_{d}^{A},$$ (1)

where $h_{d}^{\prime }\in R^{d^{\prime }}$ is the projected latent vector of node *d*, $x_{d}\in R^{d_{A}}$ is the initial vector, and $W_{A}\in R^{d^{\prime }\times d_{A}}$ is the parameter weight matrix of *A*-type nodes.

In our work, in addition to the content of two disease nodes at the start and end of the meta-path, the intermediate nodes of the meta-path, such as the human gene node and the mice phenotype node, are also important for calculating the similarity of the two diseases. For example, in our disease network containing diseases, human genes, and proteins, M1 disease → human gene → disease (DGD) and M2 disease → human gene → human gene → disease (DGGD) are two meta-paths that describe different relationships. The DGD meta-path describes two diseases associated with the same gene, while the DGGD meta-path links diseases associated with a pair of genes coding for directly linked proteins. Therefore, we can consider a meta-path as a higher-order approximation between two nodes. Furthermore, the intra-meta-path aggregation and attention mechanisms of MAGNN are applied to each meta-path, which can fully capture the contextual structure and content of the nodes of our heterogeneous biological network.

Based on the meta-path *P*, we define the target disease node as *d* and the neighbor node as $g\in N_{d}^{P}$ . In addition, we define the corresponding meta-path instance as $P\left(d,g\right)$ , and the intermediate nodes of the meta-path $P\left(d,g\right)$ as $\left\{m^{P\left(d,g\right)}\right\}=P\left(d,g\right)\backslash \left\{d,g\right\}$ .

In the intra-meta-path aggregation, we need to convert the feature vectors of all nodes of the meta-path instance into a vector $h_{P\left(d,g\right)}$ through a special meta-path encoder. The meta-path instance is treated as a set in the normal mean and linear encoders, so the information embedded in the structure of the meta-path is ignored. Relational rotation (Sun et al., 2019) provides us a better way to extract meta-path information. The relational rotation encoder is defined as follows:

$$\begin{array}{cc} o_{0} & =h_{t_{0}^{\prime }}=h_{g}^{\prime }\\ o_{i} & =h_{t_{i}}^{\prime }+o_{i-1}⊙r_{i}\\ h_{P\left(d,g\right)} & =\frac{o_{n}}{n+1}, \end{array}$$ (2)

where *R*_*i*_ is the relation between node *t*_*i*−1_ and *t*_*i*_. Given $h_{P\left(d,g\right)}$ = (*t*_0_, *t*_1_, …, *t*_*n*_), where *t*_0_ = *d* and *t*_*n*_ = *g*, let *r*_*i*_ represent the relation vector of *R*_*i*_. $h_{t_{i}}^{\prime }$ and *r*_*i*_ are both complex vectors, and ⊙ is the element-wise product.

Then, we need to define the parameterized attention (Velickovic et al., 2017) vector *a*_*P*_ of the meta-path *P* and apply the attention mechanism to perform weighted aggregation of all meta-path instances based on the meta-path *P* of the target node *d*.

$$\begin{array}{cc} e_{dg}^{P} & =LeakyReLU\left(a_{P}^{T}•\left[h_{d}^{\prime }\|h_{P\left(d,g\right)}\right]\right)\\ \alpha_{dg}^{P} & =\frac{\exp\left(e_{dg}^{P}\right)}{\sum_{s\in N_{d}^{P}}exp\left(e_{ds}^{P}\right)}\\ h_{d}^{P} & =\sigma \left(\underset{g\in N_{d}^{P}}{\sum }\alpha_{dg}^{P}•h_{P\left(d,g\right)}\right). \end{array}$$ (3)

Here, $e_{dg}^{P}$ is the importance weight of the meta-path instance $P\left(d,g\right)$ for node *d*. $\alpha_{dg}^{P}$ is the result of softmax normalization of the importance weights related to all meta-path instances of meta-path *P*, that is, the normalized attention coefficient. Then, we apply $\alpha_{dg}^{P}$ and the vector representation $h_{P\left(d,g\right)}$ of the corresponding meta-path instance to perform weighted aggregation. Finally, the vector representation $h_{d}^{P}$ based on the meta-path *P* of the target node *d* is the output through an activation function.

Through intra-meta-path aggregation, we finally obtain $h_{d}^{Pi}$ that contains all the intermediate information on the *P*_*i*_-meta-path about the target disease node *d*, where *P*_*i*_ includes four meta-paths, namely, M1 (DGD), M2 (DGGD), M3 (DGMMGD), and M4 (DGMPMGD).

One meta-path *P* can describe one composite relationship between two objects. However, the similarity between diseases is affected by many factors in bioinformatics, and diseases are not only related to genes but also related to phenotypes and proteins. Therefore, we consider multiple meta-paths and further inter-meta-path aggregation, using the attention mechanism to aggregate the potential vectors obtained from the four meta-paths to get the final node embedding so as to better capture the complex structural information on heterogeneous networks.

If the node of node type *A* has *M* meta-paths, the target node *d*_*A*_ will have a set of vectors: $\left\{h_{d}^{P_{1}},h_{d}^{P_{2}},\ldots ,h_{d}^{P_{M}}\right\}$ . Similarly, each node belonging to node type *A* will have such a set of vectors. Each meta-path vector of all nodes with node type *A* needs to be converted and averaged, respectively, as follows:

$$s_{p_{i}}=\frac{1}{\left|D_{A}\right|}\underset{d\in D_{A}}{\sum }tanh\left(M_{A}•h_{d}^{P_{i}}+b_{A}\right),$$ (4)

where meta-path $P_{i}\in P_{A}\;$ , and *M*_*A*_ and *b*_*A*_ are learnable parameters.

In a heterogeneous biological network, different meta-paths are not equally important, so we need to assign appropriate weights to different meta-paths by employing the attention mechanism to extract the information in the network more accurately.

We apply the attention mechanism to find the target node *d*_*A*_ new feature vector mixed with all meta-path information as follows:

$$\begin{array}{cc} e_{P_{i}} & =q_{A}^{T}•s_{P_{i}},\\ \beta_{P_{i}} & =\frac{\exp\left(e_{P_{i}}\right)}{\sum_{P\in P_{A}}exp\left(e_{P}\right)},\\ h_{d}^{P_{A}} & =\underset{P\in P_{A}}{\sum }\beta_{P}•h_{d}^{P}, \end{array}$$ (5)

where *q*_*A*_ is the parameterized attention vector of type node *A*. $\beta_{P_{i}}$ is the corresponding weight of meta-path *P*_*i*_ uniformly normalized to type node *A*. After calculating $\beta_{P_{i}}$ corresponding to each meta-path, the corresponding target nodes *d*_*A*_ of $h_{d}^{P}$ are weighted sum to obtain $h_{d}^{P_{A}}\;$ , and finally $h_{d}^{P_{A}}\;$ containing the information of four meta-paths.

Finally, we output the disease node embedding in the required dimension through linear transformation and non-linear activation function:

$$h_{d}=\sigma \left(W_{o}•h_{d}^{P_{A}}\right).$$ (6)

**FIGURE 2:**
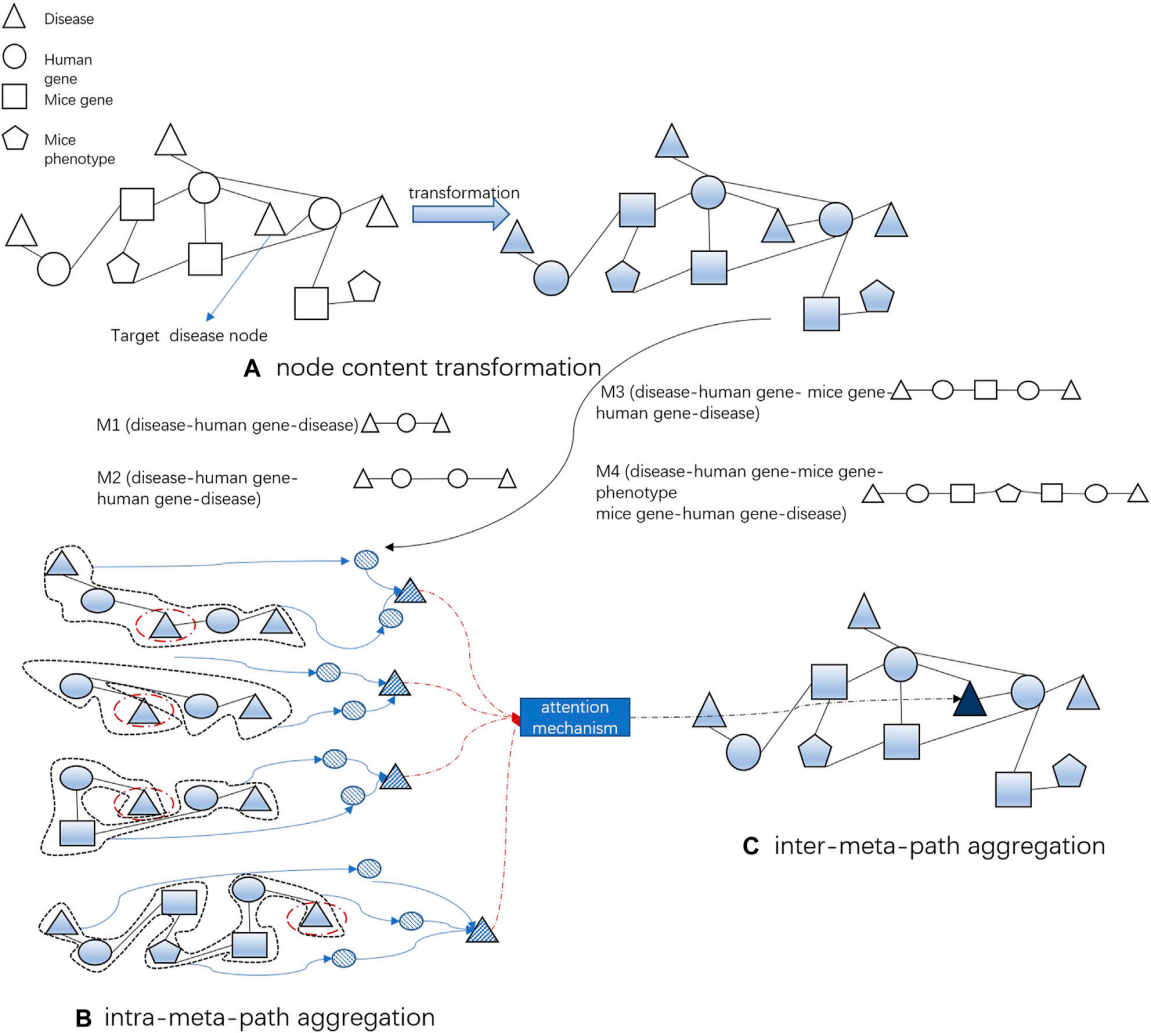
Process of DapBCH to obtain a disease node vector. **(A)** Project the different types of node features of biological networks into the same latent vector space. **(B)** Aggregate the context structure and content in the meta-path of biological networks into nodes. **(C)** Employ the attention mechanism to aggregate the potential vectors obtained from the four meta-paths to get the final node embedding.

We select four meta-paths related to disease nodes in the heterogeneous biological network so that the node embedding of the target disease can aggregate the information on multiple meta-paths through inter-meta-path aggregation. After obtaining the vector representation of disease nodes by the aforementioned method, the vector representation of gene nodes and phenotype nodes is obtained in the same way for subsequent training of the model. Figure 2 simply demonstrates the embedding generation of a single disease node.

#### 2.2.4 Disease association prediction

We apply link prediction to predict disease association. Link prediction is the analysis of existing network structures and known associations to uncover missing connections and predict possible connections. In generating the disease node embedding, we optimized our model by minimizing the loss function described in the following equation:

$$L=-\underset{\left(d_{1},d_{2}\right)\in \Omega }{\sum }\log\;\sigma \left(h_{{\;\;}^{T}}\cdot h_{d_{2}}\right)-\underset{\left(d_{1}^{\prime },d_{2}^{\prime }\right)\in \Omega^{-}}{\sum }\log\;\sigma \left({-h}_{d_{1}^{\prime }}^{T}\cdot h_{d_{2}^{\prime }}\right),$$ (7)

where $\sigma \left(\cdot \right)$ is the sigmoid function, Ω is the set of positive node pairs of known disease associations, and Ω^−^ is the complement of negative node pairs without disease associations. We optimize our model to reduce the score of unknown disease pairs and improve the score of known disease–disease associations. Since our model is an end-to-end training model, its parameters are continuously adjusted during the training process, allowing us to successfully complete the optimization task.

In a train-generated disease embedding $h_{d_{1}}$ and another disease node embedding $h_{d_{2}}$ , we calculate the probability of a disease–disease association as follows:

$$p_{d_{1}d_{2}}=\sigma \left(h_{d_{1}}^{T}\cdot h_{d_{2}}\right).$$ (8)

Finally, the disease associations are ranked using the disease association probability $p_{d_{1}d_{2}}$ predicted by the model. The higher the $p_{d_{1}d_{2}}$ association probability, the higher the likelihood that the disease pair is associated.

### 2.3 Experimental indicators

In order to evaluate the experimental results and better compare and analyze the experimental results under different experimental settings, the area under the curve (AUC) and average precision (AP) are selected as experimental indicators.

AUC refers to the area covered by the ROC curve and is used in the visual performance of the evaluation model, the AUC value in the [0, 1] interval; the bigger the AUC value is showed, the better the performance of the model.

AP refers to the area enclosed by the P–R curve, reflecting the comprehensive performance between the model’s accuracy in identifying positive examples and its coverage ability for positive examples. The AP value is in the range of [0,1]. The higher the AP value, the better the algorithm is in predicting the disease–disease association.

### 2.4 Comparison algorithm

The experimental comparison algorithms in this paper mainly include the following:

1) Metapath2vec (Dong et al., 2017): it is a classical heterogeneous network representation learning method that reconstructs the heterogeneous neighbors of a node through a single meta-path-guided random walk, which is then transmitted to the heterogeneous skip-gram model to obtain node embeddings for downstream tasks. Among other things, it obtains a single meta-path from each defined type of node by random walk, and this meta-path represents the structural information on this node. The model relies on manually pre-selecting a meta-path, so the four defined meta-paths are tested separately in the experiments, and the meta-path with the best results is demonstrated.
2) DeepWalk (Perozzi et al., 2014): it is also a pioneering algorithm. Perozzi et al. used deep learning for large-scale network analysis for the first time, combining the traditional random walk in the graph theory with the skip-gram model and using stochastic gradient descent to learn parameters, resulting in a simple and efficient network representation learning algorithm. We apply it to heterogeneous graphs by ignoring the heterogeneity of the graph structure and removing all node content features.
3) HAN (Wang et al., 2019): it uses an attention mechanism to integrate meta-path-specific node embedding, which learns a single vector representation of each node from several meta-path-based homogeneous graphs.
4) HeteWalk (Xiong et al., 2019): it is a representation learning method that generates node vectors through a heterogeneous skip-gram model, which is based on random walks guided by meta-paths and link weights. HeteWalk preserves the existing relationships by maximizing the conditional probability of each node pair appearing in the node sequence. In this case, the node sequence is created based on meta-paths.

## 3 Results

In this section, we conduct two experiments to validate our model. In the first experiment, we compare our method with four state-of-the-art network embedding methods. Through this experiment, it is confirmed that our method is more suitable than other methods for predicting disease association.

In the second experiment, we perform a series of ablation experiments. Our ablation experiments remove certain parts of the heterogeneous biological network to better understand the importance of this part of the network to the overall network. If worse result is obtained after the ablation experiment, it means that the part of the network worked. The first experiment not only evaluates the performance of each different meta-path but also evaluates the overall four meta-paths, and then compares and analyzes. The second setup is to remove mouse phenotype–gene association information in the heterogeneous network. The third experimental setup builds on the second experiment by continuing to remove the human–mice homologous gene network to generate experimental results for each model. The fourth experiment is set to delete the PPI network and compare the results with the whole network. The results of ablation experiments show the effectiveness of the model organism information and PPI information we added, and the integration of multiple different meta-paths can also help improve the prediction of disease association performance.

### 3.1 Comparison experiment and ablation experiment

First, we compare our method with four network embedding methods, namely, Matepath2vec, DeepWalk, HAN, and HeteWalk. In each comparison experiment, we conduct a 10-fold cross-validation, that is, the unconnected disease pairs (unknown associations) are first partitioned into 10 equally sized folds, among which nine folds are selected as the training data and the remaining one fold is selected as the test data. After 10 folds are completed, 10 iterations of training and verification are performed. In this way, in each iteration, different folds of data are reserved for verification, and the remaining nine folds are applied for learning. The model learnt subsequently is used to predict the data in the verification fold (Singh and Panda, 2011). For Metapath2vec, DeepWalk, HAN, and HeteWalk, we follow the original settings in their previous experiments. The window size of Metapath2vec, DeepWalk, and HeteWalk is set to 5, the walk length to 100, the number of walks per node to 40, and the negative sample count to 5. Among them, the discard rate of HAN and our proposed model is set to 0.5, the number of attention heads is set to 8, and the dimension of the attention vector in the meta-path aggregation is set to 128. For fair comparison, the embedding dimension of all models compared to 64 is set. Since Metapath2vec requires us to select only one meta-path in each experiment, we apply the M4 meta-path for it in the experiment. Each embedding model is run independently 10 times, and the average value of each model is calculated as the final prediction result. We employ the AUC and AP to compare the performance of the models. We investigate whether the similarity of known disease pairs used for optimization could be prioritized in the model as a way to generate AUC values. We report the results of each embedding model run in Table 2.

**TABLE 2:** Experimental results (%) of using our method and four network embedding methods to identify the performance of the disease association.

Method	Metapath2vec	DeepWalk	HAN	HeteWalk	MAGNN
AUC	85.68	73.21	90.87	88.23	92.62
AP	85.86	72.15	90.54	87.81	93.13

**FIGURE 3:**
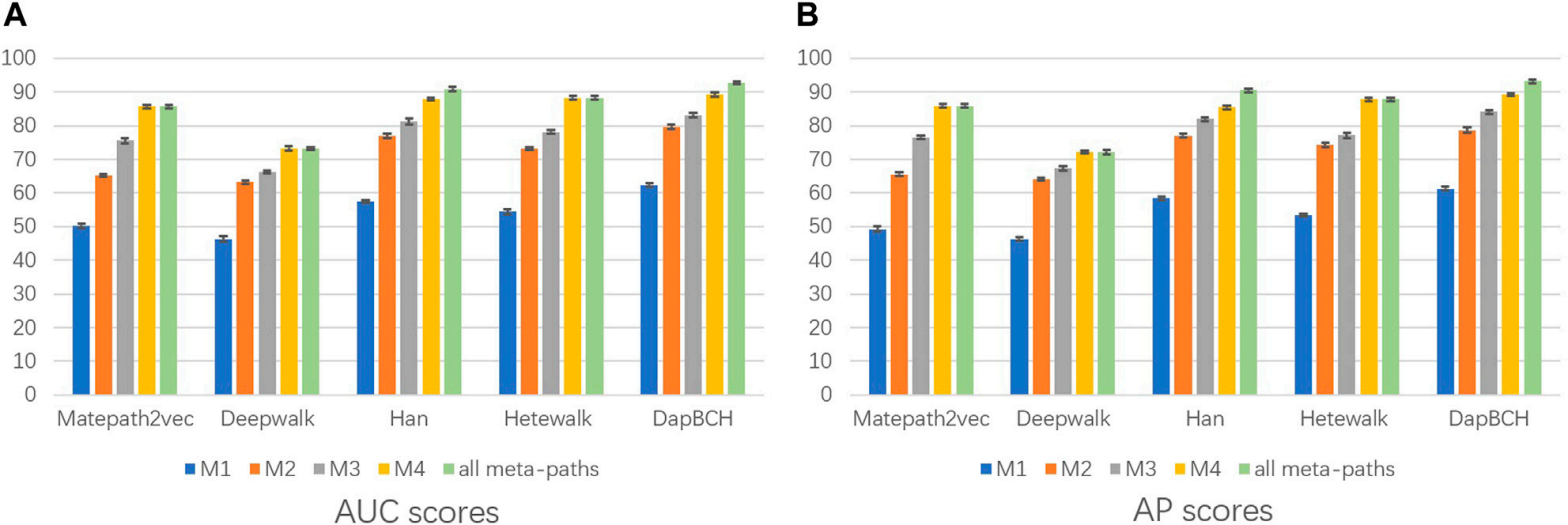
Performance of applying different meta-paths. The aforementioned figure illustrates the AUC and AP scores for disease associations predicted by four network embedding methods and our approach. The blue bars are generated by applying M1 meta-paths to each model, the orange bars indicate results from applying M2 meta-paths, the gray bars are generated by applying M3 meta-paths, the yellow bars are generated by applying M4 meta-paths, and the green bars indicate results generated by applying all meta-paths. **(A)** AUC scores. **(B)** AP scores.

**FIGURE 4:**
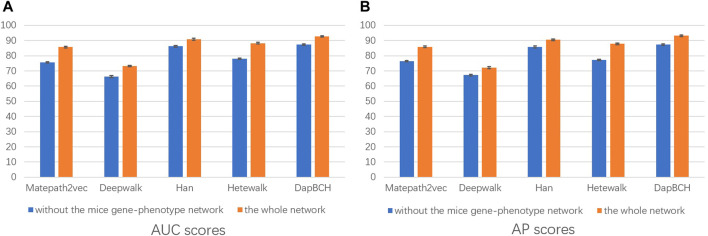
Performance of removing the mouse gene–phenotype information network prediction. The aforementioned figure illustrates the AUC and AP scores for disease associations predicted by the four network embedding methods and our methods. The blue bar represents the results of removing the mouse gene–phenotype information network, and the orange bar represents the results of the whole heterogeneous biological network. **(A)** AUC scores. **(B)** AP scores.

**FIGURE 5:**
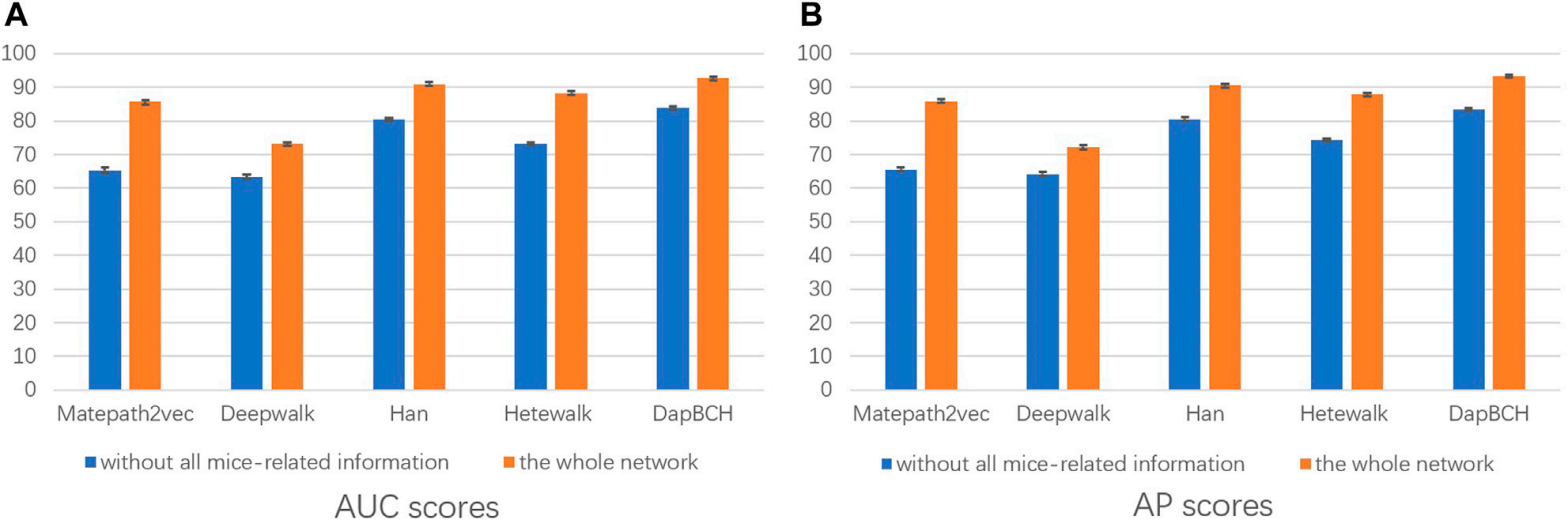
Performance of removing all mouse-related information network predictions. The aforementioned figure illustrates the AUC and AP scores for disease associations predicted by the four network embedding methods and our methods. The blue bars represent the results generated on the complete network without all mouse-related information, and the orange bars represent the results generated on the entire heterogeneous biological network. **(A)** AUC scores. **(B)** AP scores.

**FIGURE 6:**
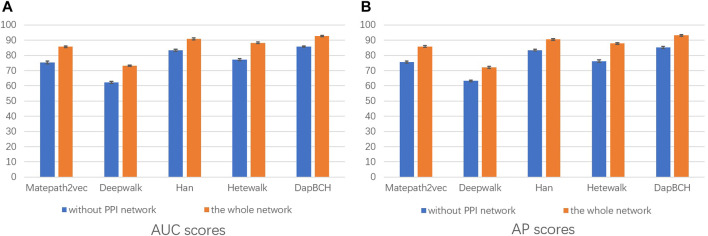
Performance of removing PPI network predictions. The aforementioned figure illustrates the AUC and AP scores for disease associations predicted by the four network embedding methods and our methods. The blue bars represent the results generated on the complete network without PPI network information, and the orange bars represent the results generated on the entire heterogeneous biological network. **(A)** AUC scores. **(B)** AP scores.

Next, we perform ablation experiments and design four different setups to confirm the effectiveness of adding biological information and to demonstrate that multiple meta-paths can improve the accuracy of predictions. The four experimental settings are as follows:

1) The first experiment not only evaluates the performance of each different meta-path but also evaluates the overall four meta-paths, and then compares and analyzes. The M4 meta-path with better performance is applied to the Metapath2Vec model in that experiment. Figure 3 shows the performance results related to the prediction of disease association when selecting each single meta-path and all meta-paths.
2) The second experimental setup removes the mouse phenotype–gene association network in the heterogeneous network we conducted, that is, only M1, M2, and M3 meta-paths are selected in the multiple meta-path model. The M3 meta-path with better performance is applied to the Metapath2Vec model in this experiment. We show the performance results of removing the mouse gene–phenotype information network *vs.* the whole network in Figure 4.
3) The third experimental setup continues the second experiment by removing the human–mouse homologous gene network, that is, only M1 and M2 meta-paths are selected in the multiple meta-path model. The M2 meta-path is applied to the Metapath2Vec model in this experiment. Figure 5 shows the performance results of the network and the complete network without all mouse-related information.
4) The fourth experiment is set to remove the PPI network, that is, only M1, M3, and M4 meta-paths are selected in the multiple meta-path model. The M4 meta-path is applied to the Metapath2Vec model in this experiment. We show the performance results for the network with PPI network information removed *vs.* the full network in Figure 6.

As in the first comparative experiment, each embedded model runs independently for 10 times in each ablation experiment.

## 4 Discussion

The experimental results showed that compared with the previous four network embedding methods, our method has improved in predicting the disease association. Figure 3 shows that our method consistently outperforms the best baseline HAN obtaining the highest AUC score of 92.62% and AP score of 93.13% under different experimental settings. From the aforementioned results, we can conclude that our method can predict the disease associations more accurately. By applying node content transformation, intra-meta-path aggregation, and inter-meta-path aggregation to generate disease node embedding, more information in heterogeneous biological networks can indeed be obtained.

In the second experiment, we perform ablation experiments. Figure 3 shows that the performance of combining multiple meta-paths is significantly improved over the performance in using only one meta-path. Among them, M4, which combines mouse-related data, has the best prediction performance in each model. Moreover, the experiments show that MAGNN and HAN receive a better score than Metapath2vec. In other words, combining multiple meta-paths by inter-meta-path aggregation gives more accurate results than selecting only a single meta-path. Figure 4 shows that when the constructed heterogeneous biological network removes the mouse gene–phenotype network, the AUC of the DapBCH model decreases by 8.86% and AP decreases by 5.87%. Figure 5 shows that when removing the mouse gene–phenotype association network and the human–mouse homologous gene network, the AUC of DapBCH decreases by 9.41% and AP decreases by 9.87%. Figure 4 and Figure 5 show that the increase in the mouse gene–phenotype association network and the human–mouse homologous gene network improves the performance results, which shows that the increased model organism information is helpful in improving the accuracy of predicting disease associations. Figure 6 shows that the results obtained by adding protein interaction information are more accurate in each model, indicating that applying the PPI network information network helps improve the model prediction performance. Thus, the ablation experiments confirm the correctness of our method that adding the mouse gene–phenotype association network, the human–mouse homologous gene network, and the PPI network, as well as selecting multiple meta-path combinations for predicting disease associations, can improve the accuracy.

## 5 Scientific verification of the predicted comorbidity

We apply the graph embedding method based on the meta-path to predict the disease association through four meta-paths in the heterogeneous biological network we constructed. We list the top 15 disease pairs with the highest similarity in one of the experimental results, excluding the known disease pairs for training, as shown in Table 3.

**TABLE 3:** Top 15 disease pairs with the highest similarity obtained from our model predicting the disease–disease association. The first column is the descending number of similarities between diseases. The second column represents disease 1 in the disease pair predicted by the model. The third column represents disease 2 of the disease pair.

Order	Disease 1	Disease 2
1	Alzheimer’s disease	Hypercholesterolemia
2	Hepatitis	Liver disease
3	Obesity	Sleep disorder
4	Bipolar disorder	Epilepsy
5	Familial combined hyperlipidemia	Hypertension
6	Asthma	Epilepsy
7	Epilepsy	Anxiety disorder
8	Systemic scleroderma	Essential hypertension
9	Systemic scleroderma	Asthma
10	Polycystic kidney disease	Congestive heart failure
11	Allergic rhinitis	Asthma
12	Mood disorder	Anxiety disorders
13	Cryoglobulinemia	Hepatitis
14	COVID-19	Severe acute respiratory syndrome
15	Male infertility	Obesity

By searching the literature (Ottman et al., 2011; Erden and Bicakci, 2012; Al-Goblan et al., 2014; Iglay et al., 2016; Su et al., 2016; Guekht, 2017; Lopez et al., 2017; Chuang et al., 2018; Newcombe et al., 2018; Oganov et al., 2019; Baradaran et al., 2020; Choi, 2020; de Lucena et al., 2020; Benhammou et al., 2021; Maciejewska et al., 2021) related to the diseases listed in the table for analysis, we found the correlation between the pathogenesis of the disease pairs. We interpret and describe some of the disease pairs in Table 3. Alzheimer’s disease–hypercholesterolemia: the pathogenesis of AD is associated with multiple complications and advanced age (Santiago and Potashkin, 2021). Schizophrenia, depression, epilepsy, sleep disorder, hypercholesterolemia, hypertension, and other pathological conditions may cause AD. Hypercholesterolemia and hypertension may impair functions such as verbal memory, verbal reasoning, and visual memory. In clinical treatment, it may help controlling these risk factors in patients diagnosed with AD (Goldstein et al., 2008). Obesity–sleep disorder: the decrease in sleep time and quality is related to the increase of weight and obesity. Sleep disorder and sleep deprivation will also worsen the development of obesity. Insomnia or other sleep disorders may cause excessive consumption of human energy, resulting in weight gain (Hargens et al., 2013).

To sum up, diseases may be related through symptoms, onset period, and other ways. The association between diseases is not accidental, and the current disease may be a risk factor for another disease. Therefore, we can realize that the common mechanism of finding comorbidity is helpful for the early intervention, prevention, and control measures and late treatment of the disease. Finally, the 15 pairs of disease listed in Table 3 can be confirmed to have some comorbidity in the relevant literature, which shows that our model is an effective method for predicting the association of diseases.

## 6 Conclusion

Understanding the association between diseases is of great significance in disease prevention, diagnosis, and treatment. In this paper, we construct a crossing species heterogeneous biological network, which consists of human genetic disease association, mouse genetic–phenotype association, human–mouse homologous genes, and protein-interacting groups, and apply the meta-path-based graph embedding method to predict the disease association. The experimental results show that compared with other previous predicting models, the AUC score of 92.62% and the AP score of 93.13% achieve the best performance. Through ablation experiments, we prove that integrating the information on model organisms into the network can improve the effectiveness of inferring the disease association. The combination of mouse genes and phenotypes achieves the best prediction results on the dataset.

Therefore, our main contributions lie in the following aspects: (1) we employ mice as a model organism to efficiently integrate its biological data into a heterogeneous bioinformatics network to predict the disease association; (2) we propose a disease association prediction model, DapBCH, which applies the graph embedding method to the aforementioned bioinformatics network, and compares its performance with four other network representation models in predicting the disease association; (3) it turns out that our integration of cross-species information (mice genes and phenotypes) can improve the predictability of disease in the network; and (4) it turns out that the multiple meta-paths and aggregated information on our model are helpful in predicting disease associations.

Our research on predicting the disease–disease association can be extended to solve practical clinical problems. By providing analytical and computational support to assess the risk of disease development and predict disease progression, we can advance clinical decision-making on possible treatments. As for future work, our method is more reliable in the homogeneous aggregation category classification with fewer disease examples and overlapping features. Therefore, we plan to combine low-cost and highly disease-sensitive heterogeneous network data to predict more specific disease associations, such as using disease–miRNA associations to predict lung cancer associations with other diseases.

## Data Availability

The original contributions presented in the study are included in the article/Supplementary Material. Further inquiries can be directed to the corresponding author.
